# Gut Microbiota Features in Young Children With Autism Spectrum Disorders

**DOI:** 10.3389/fmicb.2018.03146

**Published:** 2018-12-19

**Authors:** Lorena Coretti, Lorella Paparo, Maria Pia Riccio, Felice Amato, Mariella Cuomo, Alessandro Natale, Luca Borrelli, Giusi Corrado, Carmen De Caro, Marika Comegna, Elisabetta Buommino, Giuseppe Castaldo, Carmela Bravaccio, Lorenzo Chiariotti, Roberto Berni Canani, Francesca Lembo

**Affiliations:** ^1^Department of Molecular Medicine and Medical Biotechnology, University of Naples Federico II, Naples, Italy; ^2^Department of Physiology and Biochemistry, Faculty of Medicine and Surgery, University of Malta, Msida, Malta; ^3^Task Force on Microbiome Studies, University of Naples Federico II, Naples, Italy; ^4^Department of Translational Medical Science – Pediatric Section, University of Naples Federico II, Naples, Italy; ^5^CEINGE Advanced Biotechnologies, University of Naples Federico II, Naples, Italy; ^6^Department of Veterinary Medicine and Animal Productions, University of Naples Federico II, Naples, Italy; ^7^Department of Pharmacy, University of Naples Federico II, Naples, Italy; ^8^Istituto di Endocrinologia ed Oncologia Sperimantale, Naples, Italy; ^9^European Laboratory for the Investigation of Food-Induced Diseases, University of Naples Federico II, Naples, Italy

**Keywords:** gut microbiome, ASD, short chain fatty acids, *Faecalibacterium prausnitzii*, Bifidobacterium longum, butyrate, propionate

## Abstract

Proliferation and/or depletion of clusters of specific bacteria regulate intestinal functions and may interfere with neuro-immune communication and behavior in patients with autism spectrum disorder (ASD). Consistently, qualitative and quantitative alteration of bacterial metabolites may functionally affect ASD pathophysiology. Up to date, age-restricted cohort studies, that may potentially help to identify specific microbial signatures in ASD, are lacking. We investigated the gut microbiota (GM) structure and fecal short chain fatty acids (SCFAs) levels in a cohort of young children (2–4 years of age) with ASD, with respect to age-matched neurotypical healthy controls. Strong increase of Bacteroidetes and Proteobacteria and decrease of Actinobacteria was observed in these patients. Among the 91 OTUs whose relative abundance was altered in ASD patients, we observed a striking depletion of *Bifidobacterium longum*, one of the dominant bacteria in infant GM and, conversely, an increase of *Faecalibacterium prausnitzii*, a late colonizer of healthy human gut and a major butyrate producer. High levels of *F. prausnitzii* were associated to increase of fecal butyrate levels within normal range, and over representation of KEGG functions related to butyrate production in ASD patients. Here we report unbalance of GM structure with a shift in colonization by gut beneficial bacterial species in ASD patients as off early childhood.

## Introduction

Autism spectrum disorders (ASD) are a group of severe neurodevelopmental conditions characterized by stereotypic behavior with defective communication and social interaction deriving from a combination of genetic and environmental factors (Hallmayer et al., 2011). The influence of early life alteration in GM and its metabolites on the development of ASD symptoms is emerging. In animal models with ASD behavioral traits, GM dysbiosis was correlated to behavioral alterations, gastrointestinal tract abnormalities and immunologic alterations (Hsiao et al., 2013; Coretti et al., 2017), mimicking clinical features reported in ASD patients (de Magistris et al., 2010; Estes and McAllister, 2015). Several human pre-clinical studies have described abnormal gut bacteria in children with ASD. An unbalance in Bacteroidetes and Firmicutes phyla have been described with a decrease of Bacteriodetes/Firmicutes ratio in fecal samples of autistic children (Tomova et al., 2015). Higher abundance of *Clostridiun, Sutterella, Lactobacillus, Desulfovibrio* genera, and *Bacteroides vulgatus* was found in ASD patients with respect to neurotypical subjects (Song et al., 2004; Parracho et al., 2005; Finegold et al., 2010; Williams et al., 2011; De Angelis et al., 2013). Conversely, in some studies, *Bifidobacterium, Prevotella*, and *Akkermansia muciniphila* were found reduced in GM of ASD children (Finegold et al., 2010; Wang et al., 2011; Kang et al., 2013). More recently, reduction of Bacteroidetes in ASD young subjects and, at genus level, increase of *Collinsella, Corynebacterium, Dorea*, and *Lactobacillus* together with significant reduction of *Alistipes, Bilophila, Dialister, Parabacteroides, and Veillonella* have been reported (Strati et al., 2017). Inconsistently with previous studies, the ratio of Bacteroidetes/Firmicutes was significantly higher in Chinese children with ASD probably due to different living environment and eating habits (Zhang et al., 2018). Along with GM composition, also SCFAs levels, which are modulated by functional gut microbes, showed changes both in human patients and mouse models of ASD, possibly contributing to ASD symptoms. Butyric, propionic, acetic, valeric acids were found increased in ASD patients compared to controls and decreased in association with probiotic use (Wang et al., 2012). Abnormal levels of SCFAs in the systemic circulation may cause metabolic and neurological effects relevant to ASD (Thomas et al., 2012; Frye et al., 2016). Findings in animal models also supported this hypothesis (MacFabe et al., 2007; de Theije et al., 2014).

Several attempts have been made in elucidating GM features and to predict GM related activities in ASD, unfortunately heterogeneity in age, enrollment criteria, and methods used for GM analysis limit our knowledge in this area. In this study, we comparatively evaluated the GM composition and fecal levels of short chain fatty acids (SCFAs) in young ASD children, with a very restricted range of age, next ending from 2 to 4 years, and in age matched healthy controls.

## Materials and Methods

### Study Subjects

ASD patients (both sexes, age 2–4 years), consecutively observed at a tertiary Center for Pediatric Neuropsychiatry, were evaluated for the study.

Exclusion criteria were: ASD secondary to genetic syndromes; concomitant other neurological diseases: obesity; genetic and metabolic syndromes; immunodeficiencies; chronic diseases of the GI or respiratory tract; congenital cardiac defects; hepatic diseases; allergic diseases; food intolerances; use of antibiotics, pre-/pro- or synbiotics in the previous 4 weeks.

Patients recruitment occurred in 8 months. Twenty-five children, with suspected diagnosis of ASD, were evaluated. All children therefore carried out a full anamnestic and clinical evaluation including genetic and metabolic evaluation, eye counseling, Auditory Brainstem Response (ABR), Magnetic Resonance Imaging (MRI), electroencephalography (EEG). Children were evaluated by the pediatric neuropsychiatrist and underwent to the clinical protocol for evaluation of ASD; 20 out of 25 received a final diagnosis of ASD (first diagnosis) whereas five children did not have diagnostic confirmation or had other concurrent medical conditions (brain abnormalities, epilepsy, other genetic or metabolic diseases) and were excluded from the study. Nine out of 20 children were excluded from the study because of a recent antibiotic treatment or because of food selectivity, with dietary habits significantly different from that of the healthy controls. A total of 11 ASD patients (ASD) were recruited in this study. During the same study period 14 age matched healthy controls (HCs) visiting our Center because minor surgical procedures were enrolled (Table 1).

**Table 1:** Descriptive data of study participants.

	ASD	HCs
Subjects (*n*)	11	14
Age (months)	35 ± 5.7	35 ± 8.4
Gender (*n*)		
Male	81.8% (9)	57.1% (8)
Female	18.2% (2)	42.9% (6)
GI disorders (*n*)	2	0
DSM-5 (*n*)		
Level-1	1	–
Level-2	5	–
Level-3	5	–
ADOS (Total score)	16 ± 5.7	–

*ASD, autistic patients; HCs, neurotypical healthy controls; DSM-5 levels, Level 1 (“Requiring support”), Level 2 (“Requiring substantial support”), and Level 3 (“Requiring very substantial support”); ADOS, Autism Diagnostic Observation Schedules – total score. Age and ADOS scores are reported as mean ± SD.*

In all patients the diagnosis of ASD was made according to the Diagnostic and Statistical Manual of Mental Disorders, Fifth edition (DSM-5; American Psychiatric Association, 2013). In order to validate the ASD diagnosis according to Italian Guideline, all patients underwent to the Autism Diagnostic Observation Schedule – 2 (ADOS 2; Lord et al., 2012) and Autism Diagnostic Interview, Revised Version (ADI-R; Rutter et al., 2005) was administered to parents; moreover, the Griffiths Mental Development Scales (Gagliano et al., 2014), Vineland Adaptive Behavior Scales (VABS; Sparrow et al., 2003) and Childhood Autism Rating Scale (CARS; Schopler et al., 1988) were administered to better characterize the patients.

According to DSM-5 ASD criteria includes three levels of severity: Level 1 (“Requiring support”), Level 2 (“Requiring substantial support”), and Level 3 (“Requiring very substantial support”). The classification is split across two areas, Social Communication (SC) and Restricted and Repetitive Behaviors (RRB), mirroring core ASD symptoms. The notion of “level of support” is considered as severity of impairment, the environmental modifications required for managing day-to-day life (Kats et al., 2013). ADOS total-scores overcame the cut-off for the presence of Autism in all the evaluated subjects. Based on neuropsychiatric assessment, according to DSM-5 severity-levels, 5 patients obtained scores indicating a requiring very substantial support, 5 had a requiring substantial support, and 1 showed requiring support (Table 1).

In all study subjects we evaluated dietary habits, with the use of a 3 days diary and results evaluated with a specific software (WinFood, Medimatica Srl Colonnella (TE), Italy), and the possible presence of functional gastrointestinal disorders using a validated questionnaire (Rome III Criteria questionnaire, Italian Version) (Walker et al., 2006). All the enrolled children were from an urban area and presented similar dietary habits.

For all study subjects a stool samples (3 g) was collected in sterile vials and immediately frozen at -80°C.

The study was approved by the Ethics Committee of the University of Naples “Federico II” and all enrolled tutors give written informed consent in accordance with the sampling protocol approved by the local ethical committee (No. 312/17). Written informed consent was obtained from the parents of the participants in this study.

### V3–V4 16S rRNA Gene Sequencing and Data Analysis

Bacterial genomic DNA was extracted from frozen fecal samples using the QIAamp DNA Stool Mini Kit (Qiagen) according to manufacturer’s instructions. Extracted DNAs were checked for quality and quantity by spectrophotometric measurements with NanoDrop (ThermoFisher Scientific Inc) and stored at -20°C until processed for amplification. Sequencing samples were prepared according to the protocol 16S Metagenomic Sequencing Library Preparation for Illumina Miseq System with some modifications as previously described (Coretti et al., 2017). The V3–V4 regions of the 16S rDNA gene were firstly amplified starting from 200 ng genomic DNA with Fast Start High Fidelity PCR System (Roche Applied Science) and subsequently indexed with Nextera XT Index Kit (Illumina) in 10 cycles of PCR using KAPA HiFi HotStart System according to Illumina guidelines. After each PCR step, amplicons were purified with Agencourt AMPure XP beads (Beckman Coulter Inc.). Then, library sizes and concentrations of barcoded amplicons were assessed using Bioanalyzer DNA 1000 chip (Agilent technologies) and Qubit dsDNA BR assay kit (Invitrogen), respectively. Normalized libraries were pooled, denatured with NaOH, diluted to 10pM and combined with 25% (v/v) denatured 10pM PhiX, according to Illumina guidelines. Sequencing run was performed on an Illumina Miseq system using v3 reagents for 2 × 281 cycles.

V3-V4 16S rDNA FASTQ paired-end reads were quality filtered and assembled using PEAR (Zhang et al., 2014), retaining only those sequences showing average PHRED score≥30, read length between 400 and 500 bp and overlapping regions between mate-pair end of at least 40 nucleotides. Passing filter sequences were processed with PRINSEQ in order to obtain FASTA and quality files for further analyses (Schmieder and Edwards, 2011). Metagenomic analyses on the resulting data were conducted using Quantitative Insights Into Microbial Ecology (QIIME, version 1.9.1) (Caporaso et al., 2010). 16S rRNA gene sequencing reads were collapsed to operational taxonomic units (OTUs) using closed reference-based OTU picking method against Greengenes 16S gene database (GG, may 2013 version) (DeSantis et al., 2006) at 97% of sequences similarity; picked OTUs were classified at different taxonomic levels with the GG database. Species and Clostridium cluster classification was performed using SPINGO version 1.3 with default parameters on a representative sequence of each OTU (Allard et al., 2015). To avoid sample size biases in subsequent analyses, a sequence rarefaction procedure was applied using a maximum depth of 45,038 sequences/sample.

To assess sampling depth coverage and species heterogeneity in each sample, alpha diversity metrics were employed on rarefied OTU table using Good’s coverage, Observed species and Shannon’s diversity index. A two-sample permutation *t*-test, using 999 Monte Carlo permutations to compute *p*-value, was performed to compare the alpha diversities between sample groups. Diversity among sample communities (beta diversity) was assessed by calculating weighted and unweighted Unifrac distance matrices and then represented by two dimensional principal coordinates analysis (PCoA) plot. Statistical significance of beta diversities was assessed on weighted and unweighted UniFrac distances matrixes using ANOSIM and ADONIS methods with 999 permutations and a two-sided Student’s two-sample *t*-test. The Microbiome Regression-based Kernel Association Test (MiRKAT) (Zhao et al., 2015), a kernel-based regression method, was used to evaluate the effect of confounders by using a kernel metric constructed from weighted and unweighted UniFrac distances and adjusting for the small-sample size of the covariates. The significant associations were assessed using 9,999 permutations to verify the asymptotic *p*-value approximations.

Statistical differences in OTUs frequencies between sample groups at different taxonomic levels were assessed using nonparametric Kruskal–Wallis test taking into account False Discovery Rate (FDR) corrected *p*-values. Next, two analyses were applied on OTU tables generated by QIIME to identify key OTUs that discriminate ASD and HCs groups: Metastats comparison using the online interfaces (White et al., 2009) and LDA Effect Size analysis (LEfSe) (Segata et al., 2011). Only those OTUs reported by both methods to be significantly different between the two groups (*p* < 0.05 for Metastats, LDA > 2 and *p* < 0.05 for LEfSe) have been considered as key discriminatory OTUs.

The Phylogenetic Investigation of Communities by Reconstruction of Unobserved States (PICRUSt) was employed to predict the functional profile of the microbial communities in ASD and HC samples (Langille et al., 2013). In particular, the rarefied OTU table produced within QIIME was first corrected for multiple 16S rRNA gene copy number by using the normalize_by_copy_number.py script, then the obtained normalized OTU table was used as input in the predict_metagenomes.py script obtaining the Kyoto encyclopedia of genes and genomes (KEGG) ortholog (KOs) predictions for each sample (Kanehisa et al., 2016). KOs abundances of selected functions were compared between groups by using nonparametric Kruskal–Wallis test taking into account FDR corrected *p*-values.

Microbial interactions were investigated by generating the Spearman co-occurrence network on the basis of the relative abundances of key OTUs. The network was generated using the CoNet plugin (Faust and Raes, 2016) for Cytoscape (3.7.0, Shannon et al., 2003) by applying the following parameters: nonparametric Spearman correlation coefficients with a minimal cut-off threshold of 0.5 (*P* < 0.05, FDR corrected), null distribution generated by 1000 permutations with renormalization, and 1000 iterations for bootstraps. Network was built taking in consideration the ADOS scores, and the fecal butyrate concentrations. Autism and butyrate’s adjacent edges and connected nodes are reported as sub-networks.

### Droplet Digital PCR (ddPCR)

Absolute quantification of bacteria was examined by ddPCR using a DNA binding dye (EvaGreen) according to the manufacturer’s instructions. Species-specific primers for *F. prausnitzii* (Pfra-f: GATGGCCTCGCGTCCGATTAG and Pfra-r: CCGAAGACCTTCTTCCTCC) were designed by Chen et al. (2011). Total bacteria load in each sample was assessed using universal primers targeting the V7 region of the 16S rRNA gene (1048f: GTGSTGCAYGGYYGTCGTCA, 1194r: ACGTCRTCCMCNCCTTCCTC) (Raveh-Sadka et al., 2015). The ddPCR reaction mixture consisting of 10 μl QX200 EvaGreen ddPCR Supermix, 100 nM primers, and 0.5 ng of DNA extracted from fecal samples was mixed with 70 μl of droplet generator oil to create droplets following the manufacture’s instructions. The following thermocycling parameters were used to PCR amplify the droplets generated from each sample: one denaturation cycle at 95°C for 10 min, 40 cycles composed of 95°C for 30 s, 61°C for 30 s, and 72°C for 30 s, followed by a stabilization cycle at 98°C for 10 min. All ramp rates were at 2.5°C/s. The copies/μl output from QuantaSoft software was used to calculate the number of bacterial cells, assuming four copies for bacteria (Hospodsky et al., 2012), normalized by grams of fecal mass used for each gDNA extraction reaction (Raveh-Sadka et al., 2015).

### Determination of Fecal Butyrate and Propionate Concentration

Frozen feces weighing 1 g were diluted with saline, vortexed and centrifuged at 13,000 r.p.m. for 10 min in 2 ml tubes. The supernatants were filtered (0.45 μm) and used as the fecal extracts, which were stored at -20°C until analysis. To determine fecal butyrate and propionate concentration, frozen fecal extracts were acidified with 20 μl 85% phosphoric acid and 0.5 ml ethyl acetate, mixed, centrifuged at 14,000 r.p.m. for 1 h and extracted in duplicate. A quantity of the pooled extract containing the acidified butyrate was transferred into a 2 ml glass vial and loaded onto an Agilent Technologies (Santa Clara, CA, United States) 7890 gas chromatograph (GC) system with automatic loader/injector. The GC column was an Agilent J&W DB-FFAP (Agilent Technologies) with the length 30 m, internal diameter 0.25 mm and film thickness 0.25 μm. The GC was programmed to achieve the following run parameters: initial temperature 90°C, hold 0.5 min, ramp 20°C min^-1^, final temperature 190°C, total run time 8.0 min, gas flow 7.7 ml min^-1^ split less to maintain 3.26 p.s.i. column head pressure, septum purge 2.0 ml min^-1^. Detection was achieved using a flame ionization detector. Peaks were identified using a mixed external standard and quantified by peak height/internal standard ratio.

### Statistical Analysis

ddPCR assay and fecal butyrate and propionate measurement results in ASD and HCs samples were compared using two tailed Student’s *t*-test assuming equal variance. In this study results were considered statistically significant at *p*-value < 0.05. Significant differences were indicated in figures by ^∗^*p* < 0.05, ^∗∗^*p* < 0.01, ^∗∗∗^*p* < 0.001. ANOSIM, ADONIS and permutation *t*-test were performed using QIIME scripts, all other analyses were performed using R 3.2.0 (R Core Team, 2015). Bar plots were created by using GraphPad Prism 6.0.

### Data Deposition

The sequences reported in this study are deposited on the ‘European Nucleotide Archive’ under the accession number PRJEB29421^1^.

## Results and Discussion

### 16S Sequencing for GM Structure Evaluation of ASD Young Children

We attempted to identify the key features of GM in very young children (2–4 years of age) at first diagnosis of ASD. High-throughput sequencing analysis of bacterial 16S rRNA V3–V4 regions was conducted on fecal samples of ASD and HCs. In total, over 3.078 million of high-quality sequences (123,138.64 ± 47,651.53 reads/sample) were obtained from all 25 fecal samples, representing 4,870 operational taxonomic units (OTUs). We used a depth of 45,038 sequences/sample clustered in 4,129 OTUs. Good’s coverage > of 99% for all sequences in the two groups suggested that the majority of the phylotypes present in the samples had been identified, indicating good sequencing depth for investigation of ASD associated fecal microbiota (Table 2). Bacterial diversity within communities was significantly higher in the ASD group than in the controls, as indicated by the Shannon index, while no significant differences in number of observed species were detected between the two groups (Table 2). To date, conflicting data are available on the microbial richness of GM in patients with ASD, probably for the diverse enrollment criteria adopted worldwide and age dissimilarity of the studies participants. Therefore, the different bacterial diversity here observed in the two groups might be a distinctive factor in studies involving toddlers/preschoolers.

**Table 2:** Observed diversity and estimated phylotype coverage for 16S rRNA gene sequences at 97% similarity from NGS analysis.

Alpha diversity					Beta diversity			
Group	No. of reads	Observed species	Shannon	Good’s coverage		Student’s *t*-test	ANOSIM	ADONIS
ASD patients	495,418	920.5 ± 120.0	5.79 ± 0.53^∗^	0.993 ± 0.001	Unweighted UniFrac distances	*t* = -3.001 (*p* = 0.003)	*R* = 0.199 (*p* = 0.006)	*R*^2^= 0.074 (*p* < 0.001)
HCs	630,532	789.2 ± 255.5	4.73 ± 1.07	0.994 ± 0.002	Weighted UniFrac distances	*t* = –6.076 (*p* = 3.76E-09)	*R* = 0.368 (*p* = 0.001)	*R*^2^= 0.246 (*p* < 0.001)

*Alpha diversity indexes are reported as mean ± SD; ^∗^values are significantly different at p < 0.05.*

**FIGURE 1:**
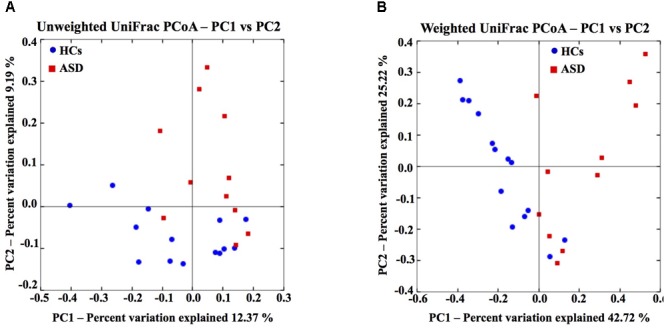
Gut microbiota structure of HCs and ASD patients. Unweighted **(A)** and weighted **(B)** UniFrac-based PCoA plot based on all OTUs of gut microbial communities (45,038 sequences/sample).

To evaluate differences in phylogenetic diversity among samples and between groups, beta diversity analysis was conducted by means of unweighted and weighted UniFrac distance metrics. Both measures, represented in two-dimentional principal coordinate analysis (PCoA) plots, showed a clustering of ASD away from the control samples (Figures 1A,B). In particular, the three different statistical methods used to compare beta diversity results showed that a more marked difference was estimated by considering weighted analysis of UniFrac distances. Results indicated that the differences in ASD and HCs microbial communities were prevalently due to changes in OTUs relative abundances (Table 2 and Figures 1A,B). Since two out of 11 patients were female and 2 patients (one male and one female) had symptoms of constipation, we evaluated the impact of these potential confounders on the differences in GM composition between the two groups. Applying MiRKAT to the weighted and unweighted UniFrac distances and adjusting for the small-sample size of the covariates, the described differences in GM composition between ASD and HCs were further confirmed (*p* < 0.01 for both weighted and unweighted UniFrac distances after gender and constipation covariate adjustments; Supplementary Figure S1).

### Phylogenetic Shift in GM of ASD Patients

**FIGURE 2:**
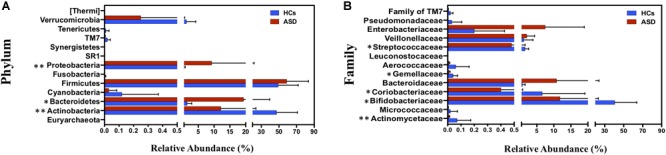
Taxonomic differences of gut microbiota between HCs and ASD groups. **(A)** Percentage distribution of all bacterial phyla identified and **(B)** percentage distribution of significant bacterial families (*p* < 0.05) between HCs and ASD. ^∗^*p* < 0.05, ^∗∗^*p* < 0.01 after FDR correction. Mean values ± SD are plotted.

At phylum level, ASD group was characterized by a marked reduction of Actinobacteria (12.18% vs. 47.30% in ASD and HCs, respectively; *p* = 0.004), and a significant increase in Bacteroidetes (19.34% vs. 1.53% in ASD and HCs, respectively; *p* = 0.04) and Proteobacteria (9.27% vs. 0.55% in ASD and HCs, respectively; *p* = 0.004; Figure 2A). Firmicutes represented the most abundant phylum both in HCs and ASD patients, without showing significant differences between the two groups. Bacteroidetes/Firmicutes ratio was significantly higher in ASD patients due to an increase in Bacteroidetes. At family level Actinomycetaceae, Coriobacteriaceae, Bifidobacteriaceae, Gemellaceae and Streptococcaceae were significantly reduced in ASD group (Figure 2B).

**FIGURE 3:**
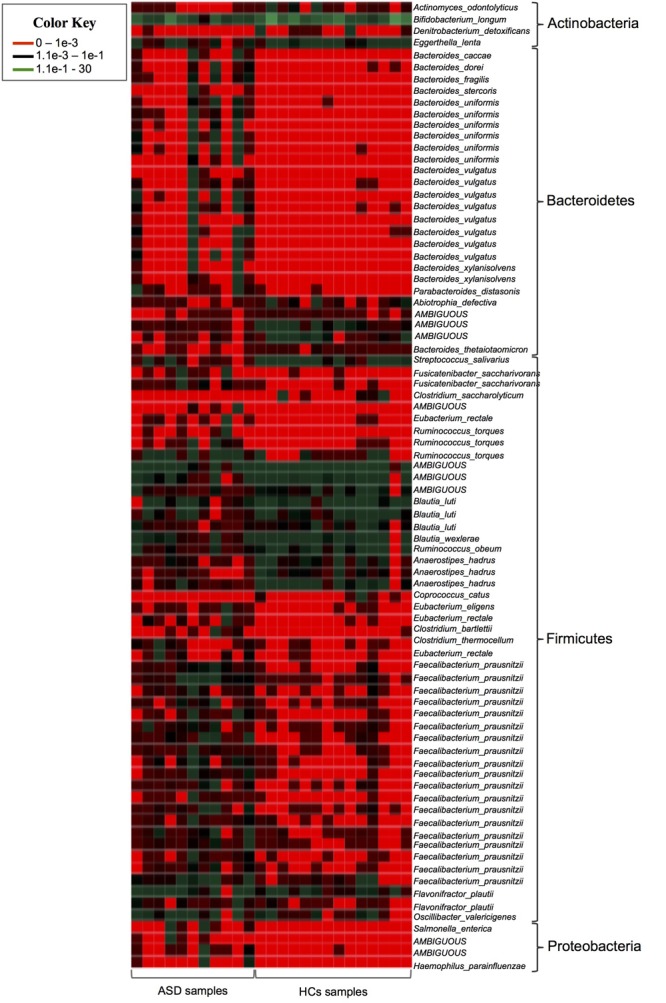
Heatmap showing the SPINGO species classification of the key OTUs with a relative abundance >0.01% (y-axis) for individual fecal samples (x-axis).

Then we identified specific phylotypes marking the differences in GM composition between the ASD and HCs groups. For 16S rRNA gene analyses a very conservative approach was applied using LefSe algorithm and Metastats comparison to detect key OTUs responsible for the differences between ASD and HCs groups (White et al., 2009; Segata et al., 2011). Among total 4,129 OTUs, we found 91 key OTUs (relative abundance >0.01%) defining the GM of ASD patients; specifically, 65/91 OTUs were found more abundant and 26/91 OTUs less abundant in ASD patients with respect to controls (Table 3). To obtain bacterial species assignment of key OTUs, sequencing data were reprocessed using SPINGO high-resolution approach (Allard et al., 2015). This analysis resolved at species level the taxonomic classification of the 91 key OTUs identifying 40 distinct bacterial species (Figure 3).

**Table 3:** Greengenes taxonomic classification and relative abundance of key OTUs defining the GM differences between ASD and HCs.

Phylum	Family	Genus	Number of OTUs	ASD	HCs
Actinobacteria	Actinomycetaceae	*Actinomyces*	1	0.002 ± 0.001	0.039 ± 0.02
	Corynebacteriaceae	*Corynebacterium*	1	0.001 ± 0.001	0.021 ± 0.007
	Bifidobacteriaceae	*Bifidobacterium (longum)*	1	6.904 ± 2.021	15.302 ± 2.943
	Coriobacteriaceae	–	1	0	0.026 ± 0.016
	Coriobacteriaceae	*Eggerthella (lenta)*	1	0.081 ± 0.031	0.408 ± 0.113
Bacteroidetes	Porphyromonadaceae	*Parabacteroides (distasonis)*	1	0.043 ± 0.028	0
	Bacteroidaceae	*Bacteroides*	22	1.606 ± 1.121	0.003 ± 0.002
Firmicutes	Aerococcaceae	–	1	0.006 ± 0.002	0.059 ± 0.027
	Streptococcaceae	*Streptococcus*	5	0.065 ± 0.03	0.704 ± 0.176
	U. Clostridiales	–	3	0.09 ± 0.05	0.1 ± 0.07
		–	2	0.16 ± 0.15	0
		*Ruminococcus*	3	0.42 ± 0.19	0.04 ± 0.02
	Lachnospiraceae	*Blautia*	10	1.06 ± 0.38	2.99 ± 0.55
		*Coprococcus*	4	0.06 ± 0.02	0.3 ± 0.08
		*Lachnospira*	1	0.04 ± 0.03	0
		*Roseburia*	1	0.06 ± 0.05	0
	Peptostreptococcaceae	–	1	0.02 ± 0.02	0
		–	18	0.97 ± 0.36	0.1 ± 0.02
	Ruminococcaceae	*Faecalibacterium (prausnitzii)*	6	0.51 ± 0.31	0.04 ± 0.01
		*Oscillospira*	3	1.21 ± 0.5	0.15 ± 0.05
Proteobacteria	Enterobacteriaceae	–	4	0.407 ± 0.368	0.002 ± 0.002
	Pasteurellaceae	–	1	0.057 ± 0.049	0

*Discriminant OTUs were identified using LEfSe algorithm and Metastats comparison (p < 0.05 for Metastats comparison and LDA > 2 with alpha < 0.05 for LEfSe). Mean ± SEM of key OTUs with relative abundance > 0.01% is reported. A more detailed table with average and SEM for each discriminatory OTU is provided as Supplementary Table S1.*

#### Low Abundance of Specific Actinobacteria Phylum Components

Among Actinobacteria, ASD patients showed a significant decrease of OTUs assigned to unclassified genus of Coriobacteriaceae, *Actinomyces, Corynebacterium* and of OTUs corresponding to *Bifidobacterium longum* and *Eggerthella lenta* (Table 3 and Figure 3). Notably, members of Actinobacteria, in particular *Bifidobacterium* spp., form a dominant fraction of the human GM, particularly in infants (Rajilić-Stojanović and de Vos, 2014). In accordance with our results, several studies reported decrease of Bifidobacteria in ASD patients (Adams et al., 2011; Wang et al., 2011; De Angelis et al., 2013; Kang et al., 2013, 2017). *Bifidobacterium* is known as a promoter of healthy status and, recently, it has been proposed as a “psycobiotic” for its ability to produce neuromodulators and influence gut–brain relationship through interaction with other commensal bacteria (Sarkar et al., 2016). The administration of *B. longum* in animal models improves anxiety, depression, and memory related behaviors (Bercik et al., 2011). In a clinical study a mixture of probiotics including *B. longum* alleviated psychological distress and urinary cortisol levels (Messaoudi et al., 2011). The severe reduction of *B. longum* in ASD young children here reported, indicates a re-assortment of key microbes of infants GM in these patients, possibly contributing to some ASD symptoms by affecting gut ecosystem and functionality.

#### Increase in Members of Gram-Negative Bacterial Phyla

Our data also showed an increase of several gram-negative bacteria with potential pathogenic features in GM of young children with ASD. Lipopolysaccharide (LPS), the major component of gram-negative cell wall, has been found increased in the serum of ASD patients and was associated with impaired social behavioral scores (Emanuele et al., 2010). LPS stimulates the secretion of proinflammatory cytokines from peripheral blood mononuclear cells and lymphoblasts of ASD children (Jyonouchi et al., 2001), probably contributing to both peripheral and brain inflammation associated with the disease (Patterson, 2011; Depino, 2013; Onore et al., 2013). In our study, among gram-negative bacteria, 22 Bacteroidetes OTUs owing to genus *Bacteroides*, mainly assigned to *B. uniformis* and *B. vulgatus* and *P. distasonis* species, were more abundant in ASD patients as well as Enterobacteriaceae and Pasteurellaceae OTUs belonging to Proteobacteria phylum (Table 3 and Figure 3).

#### Reassortment of Firmicutes Phylum Components

**FIGURE 4:**
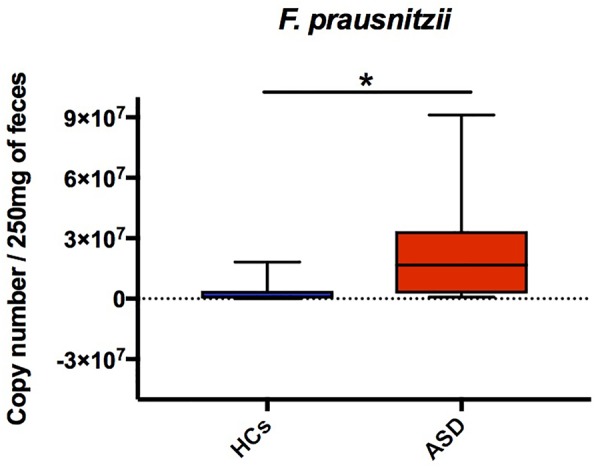
DdPCR assay showing differences of *F. prausnitzii* copy number in ASD and HCs groups. The central line within each box represents the median of the data (^∗^*p* < 0.05, Student’s *t*-test).

The majority of key phylotypes were taxonomically classified in Firmicutes phylum (58/91 OTUs; Table 3 and Supplementary Table S1 and Figure 3), depicting that, changes within Firmicutes taxa typically characterized the GM of ASD patients. *Streptococcus* OTUs were markedly reduced in ASD patients and *Clostridium* clusters were differently represented in ASD and control groups (Table 3). Notably, *Clostridium* cluster IV discriminatory OTUs assigned to unclassified genus of Ruminococcaceae, *Faecalibacterium prausnitzii* and *Oscillospira* were all significantly increased in ASD patients. SPINGO analysis showed that 13/18 OTUs of unclassified genus of Ruminococcaceae were taxonomically classified as *F. prausnitzii*, revealing that increase in relative abundance of *F. prausnitzii* marked the GM of ASD children (Figure 3). GM studies involving ASD patients have shown inconsistent results regarding the abundance of *F. prausnitzii* (Wang et al., 2011; De Angelis et al., 2013; Kang et al., 2013, 2017). These conflicting data might depend on several factors ranging from the sampling cohort to the techniques and software used to identify the GM composition. To complement and support our findings, the copy number of *F. prausnitzii* was accurately quantified using Droplet digital PCR (ddPCR) as adjunctive approach to microbiota sequencing. The analysis revealed that *F. prausnitzii* was numerically more abundant in ASD patients compared to HCs (Figure 4). The ddPCR assay also showed no significant differences in number of total bacteria in fecal samples between ASD patients and controls (data not shown). Differently from Bifidobacteria, *F. prausnitzii* is a late GM colonizer in healthy subjects and it is present at very low levels until childhood (Miquel et al., 2014). The high levels of *F. prausnitzii* in ASD patients indicate its gut premature colonization possibly at the expense of other beneficial bacteria such as *B. longum*.

### Evaluation of Fecal SCFAs Levels and Analysis of KEGG Functions Related to Butyrate Production and Mucin Degradation in ASD Patients

The higher amount of Bacteroidetes together with the altered assortment in Firmicutes taxa in ASD patients can impact on fecal SCFAs levels. SCFAs are the main end-products of bacterial fermentation in the gut and are among the most important components of microbe-host signaling, able to modulate gene expression in host cells, brain function and behavior, host energy metabolism and immune functions (Kim et al., 2016; Russo et al., 2016).

**FIGURE 5:**
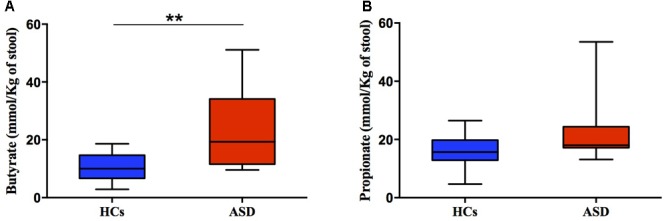
Fecal concentration (mmol/Kg) of butyric **(A)** and propionic acid **(B)** in ASD and HCs samples. ^∗∗^*p* < 0.01 for comparisons of ASD vs. HCs using Mann-Whitney test.

Here, analysis of fecal SCFAs levels, namely butyrate and propionate, revealed a trend toward an increase in ASD patients (Figures 5A,B). Butyrate and propionate fecal levels resulted within the normal range in all study subjects. But butyrate levels, even though remaining in normal limits, were significantly higher in ASD (median = 19.30 mmol/kg) compared to HCs group (median = 10.00 mmol/kg, *p* = 0.005; Figure 5A). Results suggest differences in the colonic fermentation in the two groups. In particular, data show that marked increase of *F. prausnitzii*, one of the most human butyrogenic bacteria, could account for augment of fiber fermentation capability and higher butyrate levels from gut microbes in ASD children.

**FIGURE 6:**
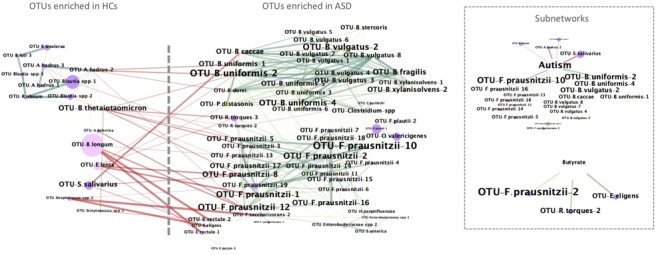
Co-occurrence network analysis of identified key OTUs and their correlation with level of ASD and butyrate. In the networks: OTUs and samples’ properties (ADOS score and fecal butyrate concentration) are depicted as nodes; an edge represents a spearman correlation with a correlation coefficient > 0.5 (green) or < -0.5 (red) that is statistically significant (FDR < 0.05); the size of each node represents the relative abundance, while the size of each label node is proportional to its degree (the number of edges and nodes connected to each node); the colors of nodes represent their classification at phylum level (pink, Actinobacteria; yellow, Bacteroidetes; violet, Firmicutes; gray, Proteobacteria). In the Supplementary Tables S2, S3 are reported the significant nodes and edges, respectively.

PICRUSt analysis was used to predict and analyze the KEGG functions involved in butyrate production (Table 4; Langille et al., 2013; Borrelli et al., 2017). Notably, the majority of the analyzed functions were enriched in ASD patients compared to controls. In particular, phosphate butyryltransferase (K00634) and acetate CoA-transferase alpha subunit (K01034), enzymatic functions involved in the final conversion of butyryl-CoA in butyrate, were both significantly higher in ASD microbial communities (Table 4). Butyrate can also be produced from mucin degradation, thus we searched for the counts of beta-hexosaminidases, a mucin-degrading enzyme. We found beta-hexosaminidase (K12373) significantly more abundant in ASD group (13,815.45 ± 3,547.44 and 3,582.57 ± 564.94 in ASD and HCs, respectively, *p* < 0.05 after FDR correction); we also observed that high abundance of the mucin-degrading enzyme in ASD patients was concurrent with the increased level of *Ruminococcus torques* which is known to be a mucin-degrading bacterium (Figure 3). Cross-feeding mechanisms in gut ecosystem have been defined to connect metabolic activity of lactic acid-producing and mucin degrading bacteria (e.g., *Bifidobacterium* and *Ruminococcus*, respectively) with butyrate producing pathways (Duncan et al., 2004; Schwab et al., 2014). Our data pinpoint the possibility that the reduction of Bifidobacteria could be counterweighed by increase of mucin degrading bacteria to sustain augment of butyrogenic *F. prausnitzii* in ASD young children. Our observations were also sustained by results obtained from co-occurrence and exclusion network analysis based on Spearman’s correlation coefficients, among microbial community structure of ASD patients and HCs, autism indices (ADOS score) and fecal butyrate levels (Figure 6). Co-occurrence network analysis revealed three ASD-enriched OTUs belonging to *F. prausnitzii, B. uniformis*, and *B. vulgatus* species as highly interconnected and potentially able to drive, in the gut of ASD patients, the presence of other 35 OTUs and generate high bacterial co-occurrence network complexity. The three mentioned ASD-enriched OTUs were also the phylotypes most negatively correlated with HCs-enriched OTUs annotated to *B. longum, E. lenta*, and *Streptococcus* OTUs, suggesting their antagonistic or mutual exclusion relationship (Figure 6). The analysis also showed few OTUs directly associated with the level of ASD and concentrations of fecal butyrate. Notably, most ASD-enriched phylotypes, such as *F. prausnitzii* and *B. uniformis*, were positively correlated to the ADOS score (*R* = 0.767 and *R* = 0.84 with *p* < 0.001 for correlation with *F. prausnitzii* and *B. uniformis*, respectively), to which HCs-enriched phylotypes were negatively correlated (Figure 6, sub-networks). Finally, 3 OTUs taxonomically classified as *F. prausnitzii, R. torques*, and *E. eligens*, were found positively associated to butyrate levels (Figure 6, sub-networks).

**Table 4:** PICRUSt KEGG Ortholog count prediction of genes codifying for key enzymes involved in butyrate production in ASD and HCs samples.

KEGG ortholog: description	ASD	HCs
K00626: acetyl-CoA C-acetyltransferase	11104 ± 1470.89	7656.86 ± 1303.27
K00074: 3-hydroxybutyryl-CoA dehydrogenase]	11688.64 ± 1977.89	12879.64 ± 1142.51
K01692: enoyl-CoA hydratase	2083.91 ± 486.13	1547.21 ± 232.23
K01715: 3-hydroxybutyryl-CoA dehydratase	7977.64 ± 2070.13	6119.93 ± 1372
K00248: butyryl-CoA dehydrogenase	11984.45 ± 2138.89	8469.43 ± 1746.3
K00634: phosphate butyryltransferase	2770.27 ± 621.97*	505.71 ± 99.14
K00929: butyrate kinase	6114.18 ± 867.86	3918.14 ± 883.67
K01034: acetate CoA-transferase alpha subunit	649.36 ± 167.35*	148.07 ± 44.15
K00016: L-lactate dehydrogenase	17782.91 ± 3128.77	24697.43 ± 2038.36

*Data are mean ± SEM. Significant differences (^∗^p < 0.05) were assessed using nonparametric Kruskal–Wallis test taking into account False Discovery Rate (FDR) corrected p-values.*

Overall we observed a reassortment of gut ecosystem in young ASD patients as off early childhood characterized by shift in colonization of gut beneficial bacterial species. The majority of patients enrolled did not suffer from GI symptoms or comorbidities, thus the observed changes in microbial colonization depended primarily on autistic disorder itself. We reported a strong depletion of *B. longum*, with *Bifidobacterium* species defined as psicobiotics (Sarkar et al., 2016), preservers of gut barrier function and immune homeostasis (Sarkar et al., 2016). Concurrently, in accordance with a previous study (Wang et al., 2013), we also detected increased levels of the mucin-degrading *R. torques* in feces of ASD children. Mucus degradation might affect intestinal epithelium permeability contributing to the development of leaky gut phenotype also described for ASD children (Li et al., 2017). Moreover, we detected significant higher levels of butyrogenic *F. prausnitzii* and butyrate in feces of ASD patients with respect to controls. Other authors also reported overrepresentation of butyrate producing bacteria in patients with major depressive disorders (Zheng et al., 2016). Butyrate has beneficial effects on human gut homeostasis (Berni Canani et al., 2011; Macia et al., 2015), host immune functions (Kim et al., 2016) and is considered a resourceful compound with promising effects in several neurologic and neuropsichiatric disorders (Bourassa et al., 2016; Stilling et al., 2016; Dinan and Cryan, 2017). Preliminary data from animal models suggested a dose-dependency of butyrate effects on the brain development and function. With positive effects on brain functions and behavior of low doses of butyrate (<500 mg/kg) and stress-like response elicited by high doses of butyrate (>1,000 mg/kg) (Gagliano et al., 2014; Takuma et al., 2014; Kratsman et al., 2016). The butyrate fecal concentration observed in ASD children in our study was within the normal range and significantly lower compared with the butyrate concentration potentially responsible for brain stress. This data strongly limits the hypothesis of a pathogenetic action elicited by butyrate in ASD. We are conscious that variations of fecal SCFAs levels in feces could depend on either poor absorption due to increased gut permeability or excessive colonic fermentation, or different exposure to environmental factors. Our data suggest that both, microbial composition (namely increase of *R. torques* and *F. prausnitzii)* and microbial predicted metabolic activity (namely increase in mucin degradation and butyrate formation enzymes) may be accountable for the observed higher level of butyrate in fecal content of ASD patients.

This study, despite limitations relative to the small number of children evaluated, encourage to further investigate the role of GM-brain axis in the critical neurodevelopmental window of early life.

## Conclusion

Our findings sustain the global alteration of GM equilibrium in ASD young children, including abnormalities in temporal colonization by *B. longum* and F. *prausnitzii*. The parallel development between GM and brain circuits, especially those required for social and emotional cognition, strongly suggest a role for GM and their metabolites in ASD symptoms and progress. In this view, it is very important to identify age-related bacterial signatures in ASD in order to develop GM-based therapies.

## Author Contributions

LCo performed microbiota analysis, statistically analyzed all data and wrote the manuscript. LP produced and analyzed butyrate and propionate data and contributed to manuscript writing. MR and GCo organized the clinical work and performed the subject’s enrolment. FA, MCo, and GCa performed ddPCR and revised the manuscript. CD produced butyrate and propionate data. MCu, AN, LB, and EB contributed to the metagenomic analysis and to the interpretation of the data. LCh conceived the investigation and contributed to the interpretation of the data. CB designed the epidemiological study, organized the clinical work, and performed the subject’s enrolment. RB conceived the investigation, designed the epidemiological study, supervised the general aspects of the work and contributed to manuscript writing. FL conceived the investigation, interpreted the data, wrote the manuscript, and critically supervised the work. All authors reviewed the manuscript.

## Conflict of Interest Statement

The authors declare that the research was conducted in the absence of any commercial or financial relationships that could be construed as a potential conflict of interest.

## Footnotes

1 https://www.ebi.ac.uk/ena/data/view/PRJEB29421
