# Corrigendum: Gut Microbiota Features in Young Children With Autism Spectrum Disorders

**DOI:** 10.3389/fmicb.2019.00920

**Published:** 2019-05-03

**Authors:** Lorena Coretti, Lorella Paparo, Maria Pia Riccio, Felice Amato, Mariella Cuomo, Alessandro Natale, Luca Borrelli, Giuseppina Corrado, Carmen De Caro, Marika Comegna, Elisabetta Buommino, Giuseppe Castaldo, Carmela Bravaccio, Lorenzo Chiariotti, Roberto Berni Canani, Francesca Lembo

**Affiliations:** ^1^Department of Molecular Medicine and Medical Biotechnology, University of Naples Federico II, Naples, Italy; ^2^Department of Physiology and Biochemistry, Faculty of Medicine and Surgery, University of Malta, Msida, Malta; ^3^Task Force on Microbiome Studies, University of Naples Federico II, Naples, Italy; ^4^Department of Translational Medical Science - Pediatric Section, University of Naples Federico II, Naples, Italy; ^5^CEINGE Advanced Biotechnologies, University of Naples Federico II, Naples, Italy; ^6^Department of Veterinary Medicine and Animal Productions, University of Naples Federico II, Naples, Italy; ^7^Department of Pharmacy, University of Naples Federico II, Naples, Italy; ^8^Istituto di Endocrinologia ed Oncologia Sperimantale, Naples, Italy; ^9^European Laboratory for the Investigation of Food-Induced Diseases, University of Naples Federico II, Naples, Italy

**Keywords:** gut microbiome, ASD, short chain fatty acids, *Faecalibacterium prausnitzii*, *Bifidobacterium longum*, butyrate, propionate

“Carmen De Caro” was not included as an author in the published article. The corrected Author Contributions Statement appears below.

The authors apologize for this error and state that this does not change the scientific conclusions of the article in any way. The original article has been updated.

## Author Contributions

LCo performed microbiota analysis, statistically analyzed all data and wrote the manuscript. LP produced and analyzed butyrate and propionate data and contributed to manuscript writing. MR and GCo organized the clinical work and performed the subject's enrolment. FA, MCo, and GCa performed ddPCR and revised the manuscript. CD produced butyrate and propionate data. MCu, AN, LB, and EB contributed to the metagenomic analysis and to the interpretation of the data. LCh conceived the investigation and contributed to the interpretation of the data. CB designed the epidemiological study, organized the clinical work, and performed the subject's enrolment. RB conceived the investigation, designed the epidemiological study, supervised the general aspects of the work and contributed to manuscript writing. FL conceived the investigation, interpreted the data, wrote the manuscript, and critically supervised the work. All authors reviewed the manuscript.

