# Supplementary material for: Gut Microbiota Features in Young Children With Autism Spectrum Disorders
Source: Front Microbiol. 2018 Dec 19;9:3146. doi: 10.3389/fmicb.2018.03146 (PMC6305749; doi:10.3389/fmicb.2018.03146)
Supplement: Supplementary file 2 [file Table_2.DOCX]

Supplementary Material

**Gut Microbiota Features in Young Children with Autism Spectrum Disorders**

**Coretti Lorena1,2,3*, Paparo Lorella4, Riccio Maria Pia4, Amato Felice1,5, Cuomo Mariella1, Natale Alessandro1, Borrelli Luca6,3, Corrado Giuseppina4, Comegna Marika1,5, Buommino Elisabetta7, Castaldo Giuseppe1,5, Bravaccio Carmela4, Chiariotti Lorenzo1,3,9, Berni Canani Roberto3,4,5,8*, Lembo Francesca3,7***

*** Correspondence:** Dr. Lorena Coretti: [lorena.coretti@tiscali](mailto:lorena.coretti@tiscali); Prof. Roberto Berni Canani: [berni@unina.it](mailto:berni@unina.it); Prof. Francesca Lembo: frlembo@unina.it


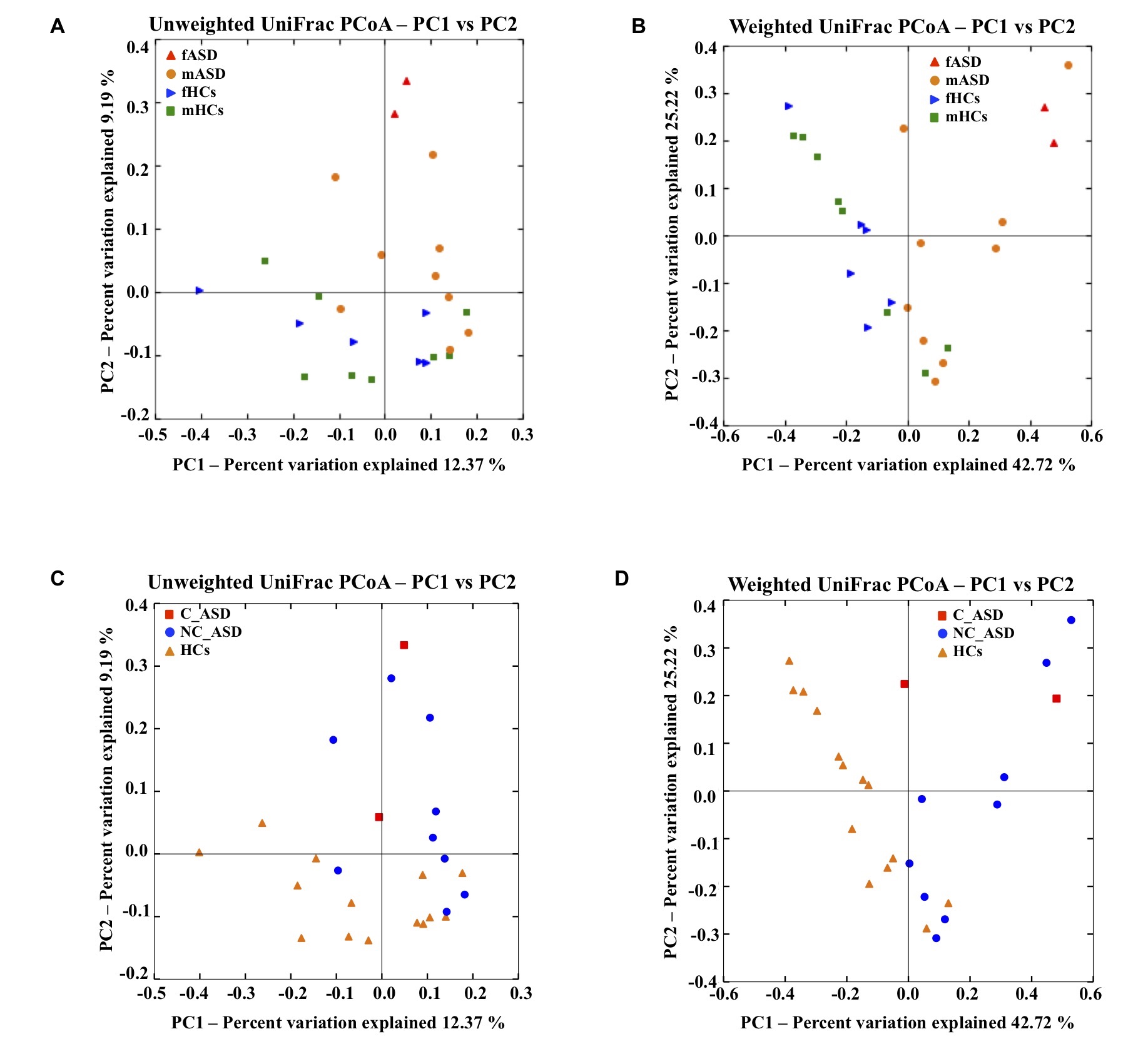


**Supplementary Figure 1**. Unweighted and weighted UniFrac-based PCoA plots on all OTUs of gut microbial communities (45,038 sequences/sample) colored for gender (A, B) and presence of constipation (C, D). Abbreviations: fASD: female ASD patients; mASD: male ASD patients; fHCs: female HC subjects; mHCs: male HC subjects; C_ASD: ASD patients with constipation and NC_ASD: ASD without constipation.
